# Measuring the complexity of general practice consultations: a Delphi and cross-sectional study in English primary care

**DOI:** 10.3399/BJGP.2020.0486

**Published:** 2021-04-07

**Authors:** Chris Salisbury, Sarah Lay-Flurrie, Clare R Bankhead, Alice Fuller, Mairead Murphy, Barbara Caddick, José M Ordóñez-Mena, Tim Holt, Brian D Nicholson, Rafael Perera, FD Richard Hobbs

**Affiliations:** Centre for Academic Primary Care, NIHR School for Primary Care Research, Population Health Sciences, Bristol Medical School, University of Bristol, Bristol.; Nuffield Department of Primary Care Health Sciences, University of Oxford, Oxford.; Nuffield Department of Primary Care Health Sciences, University of Oxford, Oxford.; Nuffield Department of Primary Care Health Sciences, University of Oxford, Oxford.; Centre for Academic Primary Care, NIHR School for Primary Care Research, Population Health Sciences, Bristol Medical School, University of Bristol, Bristol.; Centre for Academic Primary Care, NIHR School for Primary Care Research, Population Health Sciences, Bristol Medical School, University of Bristol, Bristol.; Nuffield Department of Primary Care Health Sciences, University of Oxford, Oxford; and NIHR Oxford Biomedical Research Centre, Oxford University Hospitals NHS Foundation Trust, Oxford.; Nuffield Department of Primary Care Health Sciences, University of Oxford, Oxford.; Nuffield Department of Primary Care Health Sciences, University of Oxford, Oxford.; Nuffield Department of Primary Care Health Sciences, University of Oxford, Oxford.; Nuffield Department of Primary Care Health Sciences, University of Oxford, Oxford.

**Keywords:** cross-sectional studies, Delphi technique, general practice, office visits, risk adjustment

## Abstract

**Background:**

The complexity of general practice consultations may be increasing and varies in different settings. A measure of complexity is required to test these hypotheses.

**Aim:**

To develop a valid measure of general practice consultation complexity applicable to routine medical records.

**Design and setting:**

Delphi study to select potential indicators of complexity followed by a cross-sectional study in English general practices to develop and validate a complexity measure.

**Method:**

The online Delphi study over two rounds identified potential indicators of consultation complexity. The cross-sectional study used an age–sex stratified random sample of patients and general practice face-to-face consultations from 2013/2014 in the Clinical Practice Research Datalink. The authors explored independent relationships between each indicator and consultation duration using mixed-effects regression models, and revalidated findings using data from 2017/2018. The proportion of complex consultations in different age–sex groups was assessed.

**Results:**

A total of 32 GPs participated in the Delphi study. The Delphi panel endorsed 34 of 45 possible complexity indicators after two rounds. After excluding factors because of low prevalence or confounding, 17 indicators were retained in the cross-sectional study. The study used data from 173 130 patients and 725 616 face-to-face GP consultations. On defining complexity as the presence of any of these 17 factors, 308 370 consultations (42.5%) were found to be complex. Mean duration of complex consultations was 10.49 minutes, compared to 9.64 minutes for non-complex consultations. The proportion of complex consultations was similar in males and females but increased with age.

**Conclusion:**

The present consultation complexity measure has face and construct validity. It may be useful for research, management and policy, and for informing decisions about the range of resources needed in different practices.

## INTRODUCTION

GPs in the UK report increasing pressure from a rising workload.^1^^,^^2^ The number of consultations increased by 14% between 2007 and 2014, and the mean duration of face-to-face consultations increased by 7%.^3^ Doctors’ perceptions of an increasing workload may reflect an increase in the complexity as well as number of consultations. This may be associated with an ageing population, increasing prevalence of multimorbidity and polypharmacy, transfer of activities from secondary to primary care, increasingly complex clinical guidelines, and growing policy expectations of what can be achieved within each consultation. The increasing delegation of routine tasks to other staff is also likely to increase the proportion of general practice consultations that are complex and intellectually demanding.^1^^,^^4^

To test this hypothesis, it is necessary to define and measure complexity within general practice consultations. A measure suitable for research and analysis needs to be applicable to routine electronic medical records, making it possible to explore changes in complexity over time and how consultation complexity varies in different practices, areas, and populations. A complexity measure would also be useful for resource allocation formulae, planning staffing needs, and as a case-mix variable within models to predict patient outcomes or the use of hospital and other services.

The aim of this study was to develop a valid and reliable measure of the complexity of general practice consultations that can be applied to routine medical records.

## METHOD

In this study, complex consultations are defined as those that are more difficult to conduct, challenging, multi-faceted, intensive, or time-consuming than average. This study was conducted in two stages. First, a Delphi study was conducted to agree characteristics of consultations that were perceived by GPs to be complex. Second, a valid and reliable measure was devised using cross-sectional data from a large sample of routine general practice consultations; the measure was re-validated in a separate dataset of consultations from a different year.

**Table:** How this fits in

Increasing general practice workload owing to rising consultation rates may be compounded by increasing complexity of consultations. Exploring these effects requires a valid and reliable measure of consultation complexity but there are no well-accepted measures. The authors have developed a suitable measure, starting with factors that GPs believe increase complexity and demonstrating those that are associated with longer consultations. The complexity measure presented in this study may be useful for research, management, and policy, for example in allocating resources.

### Delphi study

The research team created a list of variables that might increase the complexity of consultations based on previous literature,^5^^–^^15^ their own experience, and informal discussion with general practice colleagues. Only characteristics that were likely to be coded in routine medical records were included. Demographical factors, such as age, sex, or deprivation, were not included since the intention was to explore how the final complexity measure varied according to these characteristics.

Development and piloting of the Delphi study identified two conceptual issues. First, it was found necessary to distinguish between consultation complexity factors and patient complexity factors. Complex consultation factors were defined as problems addressed within the consultation that made it complex. However, some patients have enduring characteristics that are likely to make most of their consultations complex irrespective of the problems presented — these were defined as complex patient factors. Second, it was found that some practitioners felt that almost all their consultations were complex. Therefore, when designing the Delphi questionnaire, clinicians were asked whether each characteristic made a consultation ‘more complex than average’.

Colleagues from eight English universities were asked to each recruit five clinically active GPs to participate in the Delphi study. These doctors were asked to complete an online questionnaire in two rounds. In the first round, they were presented with 14 consultation factors and 19 patient factors and asked to indicate whether or not each factor made consultations more complex than average on a five point scale from 1 (no more complex than average) to 5 (very much more complex than average). Responders to the first-round questionnaire could add comments about individual factors or suggest additional factors that had not been included.

Factors that received strong endorsement by the panel in the first round were accepted as markers of complexity. Scores of 3 to 5 (moderate to extreme complexity) were considered to indicate endorsement of a characteristic; and a score of 1 (no more complex than the average patient) to indicate rejection. Factors that >70% of participants endorsed and <20% rejected were accepted as markers of complexity. Factors that <40% of participants endorsed and >20% rejected were not accepted. All other factors were designated uncertain and were taken forward to a second round of voting. In some cases, the wording of items was revised before the second round to provide greater clarity in the light of responders’ comments.

In the second round, participants were sent an individualised report that showed how their responses for each characteristic, and overall, compared with the median and interquartile range from other members of the panel. The report included a summary of comments from participants in round 1 about factors that had been designated uncertain. In round 2 participants were invited to vote again on the uncertain factors and on new factors that had been proposed by participants. Factors were accepted or rejected using the same criteria as for round 1. Factors that remained uncertain were rejected.

### Creating and validating a complexity measure

Read code sets were created for each of the patient and consultation complexity factors endorsed following the Delphi study. One of the authors with extensive experience of coding general practice consultations created an initial code set for each characteristic. These code sets were checked independently by two other authors (academic GPs), with discrepancies resolved by discussion or involving another author (also an academic GP). The final code sets are available at https://doi.org/10.5287/bodleian:8gq7zbb8w.

The prevalence of each characteristic was assessed based on an age–sex stratified sample from the Clinical Practice Research Datalink (CPRD) Gold database of non-temporary patients in England who were registered for any amount of time between 1 April 2013 and 31 March 2014 and had at least one face-to-face surgery consultation with a GP. Any characteristics that applied to <0.05% of consultations or patients were excluded from further consideration to simplify the measure. Frequency data were used to specify factors that had been described qualitatively in the Delphi process. For example, ‘frequent attender’ was re-specified as patients with ≥14 GP consultations in the previous year, based on the 95th centile for number of consultations.

To assess construct validity, the authors explored the independent relationship between each complexity factor and consultation duration using mixed-effects regression models of mean general practice consultation duration on patient and consultation complexity factors, with random effects for patient and practice. Consultation and patient factors were considered in separate models. Factors with a prevalence <0.05% or those that appeared to reduce the length of consultations were removed from the initial models. Remaining factors were removed in a backwards stepwise fashion using *P*<0.05 as the threshold. For a given consultation, the consultation factor applied if the topic was coded within the consultation and the patient factor applied if the consultation was with a patient with this factor.

A complex consultation was defined as one in which ≥1 complexity factors were present. The mean duration of complex and non-complex consultations was compared, and the proportion of complex consultations by age-group was described.

The described analyses of construct validity were repeated as further re-validation in a separate dataset of patients from the CPRD comprising 58 528 patients who consulted at least once between 1 April 2017 and 31 March 2018.

## RESULTS

### Delphi study

Of 41 GPs sent details of the study, 32 agreed to participate and completed the first round of the survey. Participants included 10 (31%) males and 22 (69%) females with a mean of 11.2 (median 6; range <1 to 29) years’ experience in general practice. The potential complexity factors in the first-round survey included 14 consultation factors and 19 patient factors. After the first round of the Delphi process, seven consultation factors were endorsed and none were rejected, while nine patient factors were endorsed and five were rejected. Seven consultation and five patient factors were uncertain and taken forward to round two, along with five new consultation factors and seven new patient factors suggested by panel participants. In total, there were 45 possible complexity indicators.

In round two, 30 of the 32 round-one participants (94%) completed the survey. A further 10 consultation factors and eight patient factors were endorsed, with the others being rejected or remaining uncertain, and therefore rejected. Hence, after two rounds of the Delphi survey, 34 factors were endorsed: 17 consultation factors and 17 patient factors (Tables 1 and 2).

**Table 1.** Endorsement of consultation complexity factors in two rounds of Delphi study

**Consultation variables: final wording**	**Round 1result**	**Median score^a^**	**Round two result**	**Median score^a^**	**Final status**
**Factors accepted or rejected in round one**					
Patient presents with problem of being homeless	✓	3.0	**–**	**–**	Included
Patient presents with problem which raises child protection or adult safeguarding concerns	✓	4.0	**–**	**–**	Included
Patient presents with problem which raises concerns about domestic violence	✓	4.0	**–**	**–**	Included
Consultation about learning disability/autism	✓	3.0	**–**	**–**	Included
Discussion about end-of-life issues in current consultation	✓	3.0	**–**	**–**	Included
Consultation about mental health problems	✓	4.0	**–**	**–**	Included
Multiple diagnoses or problems being managed in the current consultation	✓	3.0	**–**	**–**	Included

**Factors carried forward to round two**					
Consultation about dementia	**?**	3.0	✓	3.0	Included
Discussion about problematic drug or alcohol use in current consultation	**?**	3.0	✓	3.5	Included
Several preventive healthcare and routine monitoring tasks being conducted in same consultation	**?**	3.0	✓	3.0	Included
Procedures or minor surgery carried out in the current consultation	**?**	2.0	**?**	2.0	Rejected
Needing to prescribe many drugs in the current consultation	**?**	3.0	✓	3.0	Included
First GP consultation following a diagnosis of cancer	**?**	3.0	✓	4.0	Included
First GP consultation following a diagnosis of diabetes	**?**	3.0	✓	3.0	Included

**Factors suggested by participants and included in round 2**					
Medically unexplained symptoms raised in consultation	**–**	**–**	✓	4.0	Included
Consultation results in outpatient referral	**–**	**–**	**×**	2.0	Rejected
Consultation results in an emergency hospital admission	**–**	**–**	✓	4.0	Included
Consultation results in urgent secondary care assessment, for example, crisis team/A&E	**–**	**–**	✓	4.0	Included
Consultation about chronic pain management	**–**	**–**	✓	3.0	Included

*A&E = accident and emergency. ✓ endorsement.****?*** *uncertain. ****×*** *rejection.*

a *Scores: 1 = no more complex than the average consultation; 5 = very much more complex than the average consultation.*

**Table 2.** Endorsement of patient complexity factors in two rounds of Delphi study

**Patient variables: final wording**	**Round 1 result**	**Median score^a^**	**Round 2result**	**Median score^a^**	**Final status**
**Factors accepted or rejected in round 1**					
Homelessness (noted in the previous year)	✓	3.0	**–**	**–**	Included
Child protection/adult safeguarding (until resolved code)	✓	4.0	**–**	**–**	Included
Domestic violence (recorded in previous year)	✓	3.0	**–**	**–**	Included
Interpreter needed/no English (noted in last 3 years)	✓	3.0	**–**	**–**	Included
Learning disability/autism (noted ever)	✓	3.0	**–**	**–**	Included
Dementia (noted ever)	✓	3.0	**–**	**–**	Included
Receiving palliative care (noted ever)	✓	3.0	**–**	**–**	Included
Drug misuse/alcoholism (noted in the previous year)	✓	3.5	**–**	**–**	Included
Severe mental illness (in previous 3 years)	✓	4.0	**–**	**–**	Included
Wheelchair use (in previous 2 years)	**×**	2.0	**–**	**–**	Rejected
Recent outpatient referral	**×**	2.0	**–**	**–**	Rejected
Patient currently on warfarin	**×**	2.0	**–**	**–**	Rejected
Cancer (noted ever)	**×**	2.0	**–**	**–**	Rejected
Diabetes (noted ever)	**×**	2.0	**–**	**–**	Rejected

**Factors carried forward to round 2**					
Patient has ≥3 major long-term chronic conditions	**?**	2.0	✓	3.0	Included
Deafness (noted ever)	**?**	2.0	**?**	2.0	Rejected
Paraplegic (noted ever)	**?**	2.5	✓	3.0	Included
Blind or partially sighted (noted ever)	**?**	2.0	**?**	2.0	Rejected
Patient on immunosuppressant medication (currently)	**?**	2.0	**?**	3.0	Rejected

**Factors suggested by participants and included in round 2**					
Patient is housebound or a nursing home patient	**–**	**–**	✓	4.0	Included
Personality disorder or disruptive behaviour (noted ever)	**–**	**–**	✓	4.0	Included
Diagnostic code for ‘Medically unexplained symptoms’ entered in last year	**–**	**–**	✓	3.0	Included
Patient is morbidly obese (BMI >40)	**–**	**–**	**?**	3.0	Rejected
Frequent attender (high number of GP consultations in the last year)	**–**	**–**	✓	3.0	Included
Chronic pain recorded as a code in the last year	**–**	**–**	✓	3.0	Included
Polypharmacy (high number of drugs prescribed in the last 2 months)	**–**	**–**	✓	3.5	Included

*BMI = body mass index. ✓ endorsement* ***?*** *uncertain ****×*** *rejection.*

c *Scores: 1 = no more complex than the average consultation; 5 = very much more complex than the average consultation.*

### Creating and validating a complexity measure

Demographical characteristics of the 173 130 patients included in the 2013/2014 CPRD sample are shown in Supplementary Table S1. These patients had a total of 725 616 face-to-face consultations with a GP from 2013/2014. Supplementary Tables S2 and S3 show the prevalence of consultation complexity factors and patient complexity factors respectively, along with the final wording used to define each factor.

Factors coded in <0.05% of consultations or patients were omitted. This excluded two consultation factors: consultations about ‘medically unexplained symptoms’ and those ‘resulting in urgent secondary care assessment’ and two patient factors: ‘paraplegia’, and ‘medically unexplained symptoms within last year’. Four further factors were excluded as consultation factors but included in the modelling as patient factors: ‘palliative care’, ‘homelessness’, ‘domestic violence’, and ‘safeguarding’.

The results of the initial mixed-effects regression models of consultation and patient factors against consultation duration for 2013/2014 are shown in Table 3, with equivalent data for 2017/2018 in Supplementary Table S4. The final models, omitting variables with no significant relationship with consultation duration, include 17 factors (Table 4).

**Table 3.** Initial mixed-effects regression of consultation and patient factors against consultation duration; random effects for patient and practice^a^ (data from 2013/2014)

**Factors**	**Univariable^b^**			**Multivariable**		
	
	**Change, minutes**	**95% CI**	***P*-value**	**Change, minutes**	**95% CI**	***P*-value**
**Consultation^a^**						
Mean duration	NA	NA	NA	9.78	9.56 to 10.00	<0.001
About drug/alcohol use	4.49	3.99 to 4.98	<0.001	4.19	3.68 to 4.69	<0.001
About chronic pain	1.48	1.37 to 1.60	<0.001	0.98	0.86 to 1.10	<0.001
About dementia	1.45	0.92 to 1.98	<0.001	1.42	0.90 to 1.94	<0.001
Results in emergency hospital admission	7.81	7.12 to 8.5	<0.001	7.76	7.09 to 8.43	<0.001
About learning disability/autism	4.54	3.86 to 5.22	<0.001	3.84	3.17 to 4.52	<0.001
About mental health problems	4.06	3.92 to 4.21	<0.001	3.85	3.70 to 3.99	<0.001
≥2 diagnoses from unique Read chapters	2.99	2.87 to 3.12	<0.001	2.54	2.42 to 2.67	<0.001
≥3 unique substances prescribed	1.93	1.87 to 1.99	<0.001	1.73	1.67 to 1.80	<0.001
≥2 preventive routine tasks carried out	3.94	3.75 to 4.13	<0.001	3.73	3.55 to 3.92	<0.001
First consultation after cancer diagnosis	0.59	0.04 to 1.14	0.037	0.43	−0.11 to 0.97	0.118
First consultation after diabetes diagnosis	3.59	3.00 to 4.17	<0.001	3.05	2.48 to 3.63	<0.001

**Patient^a^**						
Mean duration of consultation	NA	NA	NA	10.02	9.79 to 10.25	<0.001
Drug/alcohol abuse in previous year	2.03	1.58 to 2.48	<0.001	1.89	1.44 to 2.33	<0.001
Chronic pain in previous year	0.87	0.72 to 1.02	<0.001	0.73	0.58 to 0.89	<0.001
Dementia (ever)	−0.78	−1.19 to −0.38	<0.001	NA^c^	NA^c^	NA^c^
Domestic violence in last year	1.46	0.40 to 2.52	0.007	1.43	0.37 to 2.49	0.008
Frequent attender (≥ 14 consultations in last year)	0.35	0.19 to 0.52	<0.001	0.01	−0.17 to 0.19	0.902
Homelessness in previous year	1.64	0.67 to 2.61	<0.001	1.36	0.39 to 2.33	0.006
Housebound or nursing home in previous 3 years	−3.72	−4.37 to −3.06	<0.001	NA^c^	NA^c^	NA^c^
No English noted in last 3 years	1.02	0.29 to 1.76	0.006	0.98	0.25 to 1.72	0.009
Learning disability/autism (ever)	0.10	−0.17 to 0.36	0.481	0.06	−0.21 to 0.33	0.654
Severe mental illness in previous 3 years	0.18	−0.36 to 0.72	0.506	−0.10	−0.64 to 0.44	0.727
≥3 long-term conditions^d^	0.45	0.36 to 0.54	<0.001	0.32	0.21 to 0.43	<0.001
Receiving palliative care (ever)	−0.58	−1.22 to 0.05	0.07	NA^c^	NA^c^	NA^c^
Personality/disruptive disorder (ever)	0.75	0.37 to 1.13	<0.001	0.51	0.12 to 0.89	0.01
Polypharmacy (≥ 9 unique substances prescribed in previous 3 months)	0.40	0.25 to 0.56	<0.001	−0.07	−0.25 to 0.11	0.447
Child protection/safeguarding in previous 3 years	−0.33	−0.69 to 0.04	0.079	NA^c^	NA^c^	NA^c^

a *Based on separate regressions for consultation and patient factors.*

b *Mixed-effect model with random intercepts for practice and patient, and a fixed effect for each patient or consultation factor at a time.*

c *Factors that had a negative relationship with consultation duration were excluded.*

d *Based on conditions included in the Cambridge Multimorbidity Score.^30^ NA = not applicable.*

**Table 4.** Final mixed-effects regression models of patient and consultation factors against consultation duration in the development and validation data sets;^a^ random effects for patient and practice

	**2013/2014 Development (*N* = 725 616 consultations)**			**2017/2018 Validation (*N* = 234 447 consultations)**		
	
	**Change, minutes**	**95% CI**	***P*-value**	**Change, minutes**	**95% CI**	***P*-value**
**Consultation factors^a^**						
Mean duration of consultation	9.78	9.56 to 10.00	<0.001	9.81	9.38 to 10.24	<0.001
About drug/alcohol use	4.19	3.68 to 4.69	<0.001	3.73	2.54 to 4.92	<0.001
About chronic pain	0.98	0.86 to 1.10	<0.001	1.00	0.79 to 1.21	<0.001
About dementia^b^	1.42	0.90 to 1.94	<0.001	NA	NA	NA
Results in emergency hospital admission	7.76	7.09 to 8.43	<0.001	4.69	3.47 to 5.92	<0.001
About learning disability/autism	3.84	3.17 to 4.52	<0.001	3.05	1.98 to 4.12	<0.001
About mental health problems	3.85	3.70 to 3.99	<0.001	3.83	3.58 to 4.08	<0.001
≥2 diagnoses from unique Read chapters recorded	2.54	2.42 to 2.67	<0.001	2.86	2.61 to 3.12	<0.001
≥3 unique substances prescribed	1.73	1.67 to 1.80	<0.001	1.82	1.69 to 1.94	<0.001
≥2 preventive/routine tasks carried out	3.73	3.55 to 3.92	<0.001	4.81	4.44 to 5.19	<0.001
First consultation after diabetes diagnosis	3.05	2.48 to 3.62	<0.001	2.39	1.60 to 3.19	<0.001

**Patient factors^a^**						
Mean duration of consultation	10.02	9.80 to 10.25	<0.001	10.03	9.60 to 10.46	<0.001
Drug/alcohol abuse in previous year^b^	1.89	1.44 to 2.33	<0.001	NA	NA	NA
Chronic pain in previous year	0.72	0.57 to 0.87	<0.001	0.75	0.51 to 1.00	<0.001
Domestic violence in last year	1.43	0.37 to 2.49	0.008	2.37	0.99 to 3.74	<0.001
Homelessness in previous year^b^	1.36	0.39 to 2.33	0.006	NA	NA	NA
No English noted in last 3 years	0.98	0.25 to 1.72	0.009	1.01	0.12 to 1.91	0.026
≥3 long-term conditions^b^	0.30	0.21 to 0.40	<0.001	NA	NA	NA
Personality/disruptive disorder (ever)^b^	0.51	0.12 to 0.89	0.01	NA	NA	NA

a *Based on separate regressions for consultation and patient factors.*

b *These variables were retained in the complexity measure despite low prevalence or lack of significant effect in the 2017–2018 model.*

The re-validation analysis used data from consultations between 1 April 2017 and 31 March 2018 and included 234 447 consultations with 58 528 independent patients. In the final model, five factors were no longer significantly associated with consultation duration (Table 4). However, the authors decided to retain these factors in their complexity measure because the factors had all been endorsed by GPs in the Delphi study, and the coefficients for duration were all positive with confidence intervals that overlapped in the development and validation data sets (see Table 3 and Supplementary Table S4). The higher *P*-values in 2017/2018 are likely to be due to the smaller total sample size.

On defining complexity as the presence of any of these factors at the consultation, 308 370 consultations (42.5%) were defined as complex in 2013/2014. The mean duration of complex consultations was 10.49 minutes, compared to 9.64 minutes for non-complex consultations, with a difference of 0.85 (95% confidence interval [CI] = 0.81 to 0.89) minutes. Equivalent analyses for the revalidation dataset in 2017 to 2018 provided very similar results, with 41.6% (97 547 out of 234 447) of consultations defined as complex. The mean duration of complex consultations in 2017/2018 was 10.32 minutes, compared to 9.70 for non-complex consultations (difference 0.62 [95% CI = 0.55 to 0.69] minutes).

**Figure 1.**
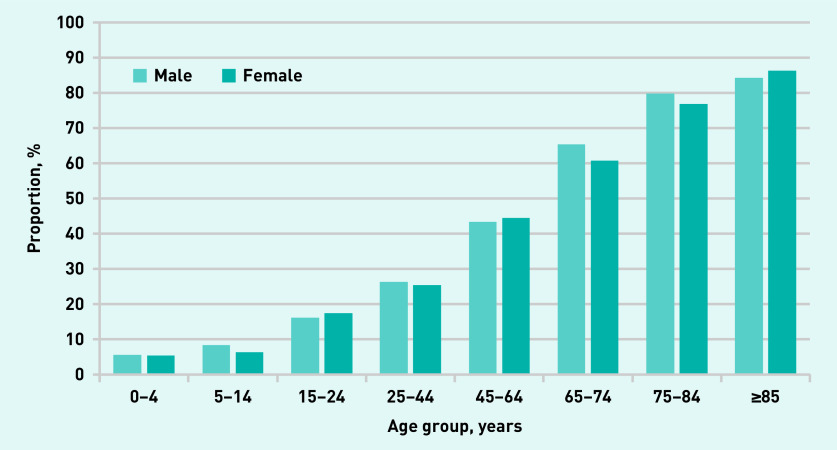
**Proportion of complex consultations stratified by age and sex.**

The proportion of complex consultations was strongly associated with increasing age, and was slightly higher in males than in females, except in patients aged >85 years (Figure 1).

## DISCUSSION

### Summary

In this article the authors have defined, created, and applied a measure of the complexity of general practice consultations which can be applied to routine electronic medical records. This measure was constructed using characteristics of patients and problems selected by a consensus process involving experienced GPs, demonstrating face validity. The measure has been validated by showing that each of the characteristics in the final selection, and the overall complexity measure, are associated with consultation duration in two independent samples of consultations.

### Strengths and limitations

This study has several limitations. The concept of complexity in consultations is nebulous, and though widely recognised by clinicians, it is hard to define.^13^^,^^16^ The present definition of complexity encompasses intellectual, emotional, and workload demands, but other definitions of complexity would lead to different measurement tools. The choice of complexity factors was based on the experience of the research team and the literature, with additional factors suggested by the GPs in the Delphi panel, but other factors could have been considered. Some factors may add complexity to consultations but are not coded within electronic medical records. In this study some factors were dropped, such as medically unexplained symptoms, which almost certainly generate complexity within consultations but are rarely coded, so inclusion would add little to the measure when used for analysis at a population level. Two variables (patients with dementia or patients who are housebound) had a statistically significant negative association with consultation duration. In post-hoc analysis it was found that these characteristics were associated with more consulting time over a whole year, resulting from a higher number of consultations that are shorter than average.

The development of the complexity measure was conducted in England, and factors that cause consultation complexity may differ in other countries, for example insurance status in the US.^13^^,^^14^ The complexity measure developed here was based on a sample of consultations taken 6 years ago. This was deliberate to create a baseline against which to assess changes in complexity over time in a subsequent article. However, in this study the authors have revalidated the findings in a more recent dataset (2017/2018) and this analysis largely confirmed the present findings.

The authors recognise that mean duration of consultations is not a gold standard for complexity, since the length of a consultation is only partly related to complexity and not all complex consultations are lengthy. However, it was the best and simplest (while imperfect) proxy available within routine medical records. The purpose of the cross-sectional analysis was not to derive a model to predict consultation duration, but to provide evidence for the construct validity of the present complexity measure by showing a positive association with a variable (duration) that the authors hypothesised would be related to it. The analysis fulfilled the present aims by confirming: that each of the included complexity factors was independently associated with longer consultations; that a measure defined as the presence of ≥1 of these factors was discriminating, with complex consultations being on average 9% longer than non-complex consultations; and that these findings were robust when repeated in a different data sample. Though the complexity measure is useful as a binary ‘complex/non-complex’ variable, the authors do not propose combining the factors to create a cumulative score (see statistical note in Supplementary Box 1).

The present measure is reliable in that it is based on objective analysis of medical records and defined code sets for complexity factors, unlike measures that require subjective judgements.^4^^,^^7^^,^^13^^,^^17^ Basing the measure on the views of practising GPs and assessing the relationship with consultation duration provides evidence of face and construct validity respectively.

Further validation exercises could explore the relationship between the present complexity measure and other variables, such as practitioners’ self-assessment of the complexity of a sample of consultations. Future research should also explore the relationship between complexity and risk prediction models for healthcare utilisation. The authors anticipate some, but not complete, overlap.^14^ It is likely that different tools will be best at predicting different outcomes and measures should be used in combination to understand population healthcare needs.^18^

### Comparison with existing literature

The presented research builds on previous research. Two studies^4^^,^^7^ and an online survey^2^ have asked primary care clinicians to record the complexity of their consultations subjectively, for example using a five-point scale from very simple to very complex, while another study quantified the number and range of problems discussed within consultations.^19^ Three studies have asked GPs about features that make patients complex, and the present authors build on this by considering aspects of consultations as well as patients.^12^^,^^14^^,^^15^^,^^20^ A few previous authors have devised case-mix measures applicable to primary care, but these have either not taken account of clinicians’ perceptions of the complexity of different factors^21^^–^^24^ or not been designed for analysis of routine medical records.^13^^,^^17^

There is some overlap between measures of complexity and case-mix measures such as Adjusted Clinical Groups,^25^ Rx-Risk^26^ and the Charlson score,^27^ which have been designed to predict health outcomes, resource utilisation, or mortality. These case-mix measures are based on combinations of diagnostic information, medication data and/or demographic factors but do not account for social, behavioural, or other psychological factors,^11^ which often create the greatest demands on GPs within consultations^12^^,^^15^^,^^16^^,^^20^ and are captured by the present complexity measure

### Implications for research and practice

This article describes a valid and reliable measure of the complexity of GPs’ consultations. In future research the authors plan to explore the complexity of consultations in different settings and populations, and how complexity has changed over time. This may be relevant to the development of resource allocation formulae. The current UK formula for allocating payments to primary care takes account of the number of expected consultations based on characteristics of the practice population, but not the complexity of those consultations.^28^ Practices that have a high proportion of complex consultations may need a different mix of staff than practices with few complex consultations. There is growing interest in creating population health management systems by linking health and social care datasets to understand current and future health and care needs.^29^ Use of a complexity measure may support this aim, providing greater nuance and understanding by taking account of the different workforce, workload, and resource implications of consultations with different levels of complexity.

## References

[1] British Medical Association (2020). Pressures in general practice. BMA.

[2] (2019). Pulse 2019 review: GP workload laid bare. Pulse.

[3] Hobbs FDR, Bankhead C, Mukhtar T (2016). Clinical workload in UK primary care: a retrospective analysis of 100 million consultations in England, 2007–14. Lancet.

[4] Gemmell I, Campbell S, Hann M, Sibbald B (2009). Assessing workload in general practice in England before and after the introduction of the pay-for-performance contract. J Adv Nurs.

[5] Salisbury C, Procter S, Stewart K (2013). The content of general practice consultations: cross-sectional study based on video recordings. Br J Gen Pract.

[6] Baird B, Charles A, Honeyman M (2016). Understanding pressures in general practice.

[7] Mercer SW, Fitzpatrick B, Gourlay G (2007). More time for complex consultations in a high-deprivation practice is associated with increased patient enablement. Br J Gen Pract.

[8] Vedsted P, Christensen MB (2005). Frequent attenders in general practice care: a literature review with special reference to methodological considerations. Public Health.

[9] Deveugele M, Derese A, van den Brink-Muinen A (2002). Consultation length in general practice: cross sectional study in six European countries. BMJ.

[10] Royal College of General Practitioners (2013). The 2022 GP. Compendium of evidence.

[11] Rosen AK, Reid R, Broemeling AM, Rakovski CC (2003). Applying a risk-adjustment framework to primary care: can we improve on existing measures?. Ann Fam Med.

[12] Grant RW, Ashburner JM, Hong CS (2011). Defining patient complexity from the primary care physician’s perspective: a cohort study. Ann Intern Med.

[13] Peek CJ, Baird MA, Coleman E (2009). Primary care for patient complexity, not only disease. Fam Syst Health.

[14] Hong CS, Atlas SJ, Ashburner JM (2015). Evaluating a model to predict primary care physician-defined complexity in a large academic primary care practicebased research network. J Gen Intern Med.

[15] Loeb DF, Binswanger IA, Candrian C, Bayliss EA (2015). Primary care physician insights into a typology of the complex patient in primary care. Ann Fam Med.

[16] Safford MM, Allison JJ, Kiefe CI (2007). Patient complexity: more than comorbidity. the vector model of complexity. J Gen Intern Med.

[17] Pratt R, Hibberd C, Cameron IM, Maxwell M (2015). The Patient Centered Assessment Method (PCAM): integrating the social dimensions of health into primary care. J Comorb.

[18] Safford MM (2015). The complexity of complex patients. J Gen Intern Med.

[19] Procter S, Stewart K, Reeves D (2014). Complex consultations in primary care: a tool for assessing the range of health problems and issues addressed in general practice consultations. BMC Fam Pract.

[20] Webster F, Rice K, Bhattacharyya O (2019). The mismeasurement of complexity: provider narratives of patients with complex needs in primary care settings. Int J Equity Health.

[21] Tonelli M, Wiebe N, Manns BJ (2018). Comparison of the complexity of patients seen by different medical subspecialists in a universal health care system. JAMA Netw Open.

[22] Halter M, Joly L, de Lusignan S (2018). Capturing complexity in clinician case-mix: classification system development using GP and physician associate data. BJGP Open.

[23] Katerndahl D, Wood R, Jaén CR (2011). Family medicine outpatient encounters are more complex than those of cardiology and psychiatry. J Am Board Fam Med.

[24] Katerndahl DA, Wood R, Jaén CR (2010). A method for estimating relative complexity of ambulatory care. Ann Fam Med.

[25] Weiner JP, Starfield BH, Steinwachs DM, Mumford LM (1991). Development and application of a population-oriented measure of ambulatory care case-mix. Med Care.

[26] Sloan KL, Sales AE, Liu CF (2003). Construction and characteristics of the RxRisk-V: a VA-adapted pharmacy-based case-mix instrument. Med Care.

[27] Charlson ME, Pompei P, Ales KL, MacKenzie CR (1987). A new method of classifying prognostic comorbidity in longitudinal studies: development and validation. J Chronic Dis.

[28] DH Financial Planning and Allocations Division (2011). Resource allocation: weighted capitation formula.

[29] NHS England Population Health and the Population Health Management Programme. https://www.england.nhs.uk/integratedcare/building-blocks/phm/.

[30] Payne RA, Mendonca SC, Elliott MN (2020). Development and validation of the Cambridge Multimorbidity Score. CMAJ.

